# Turning date palm waste into carbon nanodots and nano zerovalent iron composites for excellent removal of methylthioninium chloride from water

**DOI:** 10.1038/s41598-020-73097-x

**Published:** 2020-09-30

**Authors:** Munir Ahmad, Mutair A. Akanji, Adel R. A. Usman, Abdullah S. F. Al-Farraj, Yiu Fai Tsang, Mohammad I. Al-Wabel

**Affiliations:** 1 Soil Sciences Department, College of Food and Agricultural Sciences, King Saud University, P.O. Box 2460, Riyadh, 11451 Kingdom of Saudi Arabia; 2 Department of Soils and Water, Faculty of Agriculture, Assiut University, Assiut, 71526 Egypt; 3 Department of Science and Environmental Studies, The Education University of Hong Kong, Hong Kong, China

**Keywords:** Environmental sciences, Materials science

## Abstract

Novel carbon nanodots (nCD-DBC) and nano zero-valent iron composites (nZVI-DBC) were synthesized using date palm waste-derived biochar (DBC). The synthesized materials were analyzed for chemical and structural composition by using FTIR, SEM, XRD, and TGA, and evaluated for their methylthioninium chloride dye (MB) removal efficiency from contaminated aqueous solutions. pH 7.0 was found optimum for the highest MB removal in sorption batch studies. Kinetics sorption of MB onto the sorbents was best described by pseudo-second-order (*R*^*2*^ = 0.93–0.99) and Elovich models (*R*^*2*^ = 0.86–0.97) implying that sorption was being controlled by chemisorption. Langmuir model predicted maximum sorption capacities for nCD-DBC, nZVI-DBC, and DBC were 1558.66, 1182.90, and 851.67 mg g^−1^, respectively, which correlated with the results of kinetics sorption. Likewise, nCD-DBC yielded the highest partition coefficient (7067 mL g^−1^), followed by nZVI-DBC (1460 mL g^−1^), and DBC (930 mL g^−1^). Post-sorption XRD, FTIR, and SEM analyses depicted the binding of MB onto the sorbents. It was suggested that electrostatic interactions, π–π electron donor-accepter interactions, degradation, and diffusion were responsible for MB removal by the synthesized materials. Therefore, the nCD-DBC, nZVI-DBC, and DBC can potentially be used for scavenging MB dye from contaminated aqueous solutions.

## Introduction

Rapid industrialization and urbanization have resulted in serious threats to the ecosystem and humanity that require immediate consideration. Expansion in textile, leather, paper, pharmaceutical, personal care products, and food industries has greatly increased the utilization of dyes^1,2^. It has been estimated that currently > 100,000 dyes are available commercially with an annual production of 7 × 10^5^ tons^3^. Dyes are not easily biodegraded, and their presence in water bodies reduces light penetration; which affects photosynthesis by aquatic plants. Prevalence of dyes in wastewater may lead to excessive consumption of dissolved oxygen as a result of biological and chemical changes of the components of wastewater, which further damages aquatic life. Additionally, the prevalence of dyes in drinking water might result in cancer, mutagenic disorders (nucleotide mutation in deoxyribonucleic acid (DNA)), dermatitis, allergy, and skin irritations in animals and humans. Among the various dyes used currently, methylthioninium chloride, also known as methylene blue (MB) is the most frequently used dye for coloring of paper, cotton, wood, silk, hair, and wool. It is a basic cationic compound composed of nitrogen and sulfur atoms^3,4^. It is considered a highly toxic dye and if humans are exposed to it for longer period of time, it may lead to a state of mental disorder, abdominal pain, high blood pressure, nausea, vomiting, jaundice, increased heartbeat, quadriplegia, and tissue necrosis^5^. Therefore, limiting the introduction of MB into water bodies through industrial wastewater streams, as well as the removal of prevailing MB from wastewater is obligatory for sustainable environmental health.

Various technologies, for instance, ultrafiltration, chemical coagulation, photocatalytic degradation, electrochemical process, degradation by oxidation, and adsorption are commonly used for MB dye removal from contaminated wastewater streams before it is discharged into various natural environmental matrices. Furthermore, combined operations that use adsorption and electro-chemicals have also been employed for decoloration of wastewater contaminated with MB dye^2,6–9^. However, a majority of the aforementioned technologies are impracticable due to their higher operating costs, complexity, or inefficiency, and therefore, adsorption is considered to be a widely accepted technology due to its efficacy, simplicity, and cost-effectiveness^2,10^.

Selection of an appropriate adsorbent with higher efficiency and economic value is of critical importance. Activated carbon, biochar, metal-oxide nanoparticles, lignocellulosic bio-matter, sewage sludge, coconut tree sawdust, coir pith, banana pith, and various other adsorbents have been used for MB removal by various researchers^11,12^. However, majority of the traditionally used adsorbents are either expensive or inefficient. For instance, despite of large specific surface area and highly porous structure, the wide spread application of activated carbon is limited owing to higher costs of generation and regeneration. Likewise, biochar is considered to be an ideal candidate for sorption of a range of environmental pollutants owing to large surface area, suitable surface functional groups, and resistance toward decomposition^13^. However, depending upon the biomass type and pyrolysis conditions, the performance of biochar does not remain consistent. Similarly, the use of iron-metal based adsorbents to remove MB dye from wastewater has become obsolete due to its corrosive nature. Recently, the nano metal-oxide based adsorbents such as carbon nanotubes, nano-diamond, nano-TiO_2_, and nano-ZnO, have been used for dye degradation and removal; however, their toxicity has been reported widely^14^. Therefore, based on the aforementioned facts, development of composite adsorbents by combining porous carbonaceous materials such as biochar with nano-materials could provide multiple benefits such as cost-effectiveness, environment friendliness, efficiency, and ease in operation.

Nano zerovalent iron (nZVI) is highly reactive, cheaper, and strong reductive agent that can potentially remove a range of pollutants from water including MB dye; however, due to fine size and higher reactivity, nZVI particles are unstable in aqueous media. Thus, its applicability is restricted due to agglomeration and prompt oxidation^15,16^. Hence, supporting nZVI with porous carbonaceous materials such as biochar may develop a stabilized magnetic adsorbent for practical application. Therefore, compositing nZVI into biochar can improve the sorption capacity of biochar by many folds on one hand, and increase the stability and nZVI particles distribution on the other hand^15^. This way of combining nZVI and biochar would provide synergistic effects. Likewise, biochars can also be combined with nanodots to develop nano-biochar based adsorbents with improved characteristics and performance. Carbon nanodots are insoluble in water, non-toxic, cheaper, eco-friendly, and possess a strong fluorescence nature; they have thus found different applications, including photocatalyst, energy, bioimaging, and sensors^17^. Carbon nanodots are nanostructured and are mainly composed of oxygen, hydrogen and carbon. However, up to the best of our knowledge, no reports are available for the synthesis of carbon nanodots from biochar and their application for MB dye removal. Therefore, we hypothesized that turning date palm waste-derived biochar into carbon nanodots and nZVI-composites may develop efficient, cheaper, and green sorbents for the effective scavenging of MB dye from wastewater. In such a combination, biochar was contributed by the date palm leaf waste biomass, which was designed further to develop nZVI-composited biochar and carbon nanodots. The produced adsorbents were characterized for variations in their surface, structural, physio-chemical, and morphological compositions. Further, the efficiency of the produced sorbents for MB removal was evaluated via kinetics, isotherm, and pH adsorption batch studies, and the sorption mechanisms were explored using different kinetics and equilibrium sorption models.

## Methodology

### Chemicals

Ferrous sulfate heptahydrate (FeSO_4_·7H_2_O, > 99%) and sodium borohydride (NaBH_4_, > 98%) were purchased from Merck (Darmstadt, Germany). Ethanol, acetic acid (H_3_COOH), and sodium hydroxide (NaOH) were obtained from Fisher Scientific Co. (Springfield, NJ), while ammonium acetate was purchased from Loba Chemie, India. Reagent grade chitosan (low molecular weight) and MB dye (basic blue 9, C_16_H_18_CIN_3_S) were purchased from Sigma Aldrich. Deionized water of 18.2 MΩ cm^−1^ resistivity, purified by Milli-Q, Germany, was used in all the experiments.

### Sorbent synthesis

#### Biochar production

Date palm tree waste including leaflets and rachis was collected from date palm orchards around Riyadh city, Saudi Arabia. Leaves were separated, chopped into a length of < 5 cm, after washing with water. The chopped leaves were dried, grinded, passed through a sieve of 0.6 mm, and labeled as DF. A known mass of DF was put in crucibles with loose covers and kept in a tube furnace (Carbolite, type 3216, UK) at 600 °C for 3 h with pyrolysis rate of 5 °C per min under an oxygen-limited environment. After completion of pyrolysis process, the material was left for cooling. Next day, the produced biochar (DBC) was washed by using deionized water, then dried and milled at 800 rpm for 20 min in a Fritsch Pulverisette 7 ball-mill (Germany).

#### Fabrication of carbon nanodots

Carbon nanodots were produced by modifying the procedure reported by Li et al.^18^. Five grams of the milled DBC along with two g of citric acid were suspended in 100 mL of deionized water, followed by stirring for 3 h on a magnetic stirrer. Thereafter, 2.0% H_3_COOH solution containing 1.0 g of chitosan was added slowly under continuous stirring. Subsequently, 100 mL of NaOH (1.2%) was added into the suspension, followed by 30 min of gentle stirring. After complete homogenization, the suspension was heated at 180 °C for 2 h in airtight containers. The carbon nanodots thus derived from DBC were separated from the suspension, washed with methanol and deionized water, and labeled as nCD-DBC.

#### Synthesis of nZVI composited biochar

The nZVI composited DBC was synthesized through iron reduction by using NaBH_4_ in oxygen-less environment^13^. Briefly, a solution of FeSO_4_·7H_2_O (1.0 M) was prepared in diluted ethanol (75% ethanol in deionized water). About 100 mL of prepared FeSO_4_·7H_2_O solution was taken into a beaker and 5.6 g of the milled DBC was suspended into it. The pH of the suspension was set at 5.0 and stirred for 3 h. After purging with N_2_ for another 2 h, 100 mL solution of NaBH_4_ (2.0 M) was poured into the suspension drop-by-drop under continuous N_2_ supply and stirring. Afterwards, a solution containing 1.12 g of chitosan in 2.0% H_3_COOH was poured into the suspension under continuous stirring. Thereafter, 100 mL of NaOH (1.2%) solution was added. The supply of N_2_ was stopped, the container was air-tightened and kept overnight. Next day, the precipitates were separated from the solution, washed several times with ethanol, vacuumed dried and labeled as nZVI-DBC.

### Characterization

#### Yield, proximate, and chemical analyses

Equation 1 was used to calculate the percent yield of the biochar produced:

1 $$\text{Yield }\left(\mathrm{\%}\right)=\frac{\text{Weight of DF}-\text{Weight of DBC}}{\text{Weight of DF}} \times 100$$

The proximate analyses of the biomass and the produced materials were conducted according to the standard procedures^19^. Ammonium acetate extraction method was used to determine the cation exchange capacity (CEC) of the materials^20^. pH and electrical conductivity (EC) of the materials were determined in a 1:10 (w/v ratio of materials to deionized water) suspension. The elemental composition of the materials was determined using a series II, PerkinElmer, CHNSO elemental analyzer, USA. The particle size distribution of synthesized sorbents was determined via Malvern Mastersizer 2000 laser particle size analyzer.

#### Ultimate analyses

The materials produced in this study were observed under an EFI S50 Inspect, scanning electron microscope (SEM) to record information on their structural changes and surface morphology as caused by physiochemical changes. Samples were coated using gold nano particles after spreading on carbon tapes and images were captured between magnification rate of ×2000–3000. The stability of the materials against thermal treatments was investigated via Thermogravimetric analyzer (Model: DTG-60H, Shimadzu, Japan). The functional groups and structural composition of the materials produced were examined with the aid of a Bruker Alpha-Eco ATR-Fourier transform infrared spectroscopy (FTIR: Bruker Optics Inc.). The changes in mineralogical composition were assessed using a MAXima_X XRD-7000 (Shimadzu, Japan) X-Ray diffractometer. With the aid of TriStar II 3020 Micromeritics (USA), the pore diameter, total pore volume, and surface area were examined with Brunauer–Emmett–Teller (BET) procedure.

### Sorption experiments

#### Effect of solution pH on MB sorption

The effect of solution pH on the sorption of MB was investigated in sorption batch studies. Different solutions of pH 3.0, 5.0, 7.0, and 10 were prepared in deionized water with initial MB dye concentration of 200 mg L^−1^. A 30 mL solution of MB dye was taken into a polypropylene tube and 21 mg of the sorbent was suspended into it. Each sorbent and blank (without sorbent) were replicated thrice. The sorbent-MB suspension was shaken for 24 h at 150 rpm under room temperature (23 ± 2 °C). The solution was separated from the sorbent via centrifugation and filtered through Whatman-42 filter papers. The equilibrium MB concentration in solution was determined by using Lambda EZ 150, UV/VIS spectrophotometer (PerkinElmer, US).

Equation 2 was used to estimate the amount of MB adsorbed onto per unit of mass of the sorbent^21,22^:

2 $${q}_{e}=\left[\frac{{C}_{o}- {C}_{e}}{m}\right]\times v$$

where, *C*_*o*_ = initial MB concentration (mg L^−1^), *C*_*e*_ = equilibrium MB concentration (mg L^−1^), *m* = mass of the adsorbent (g), *v* = the volume of solution (L).

#### Kinetic sorption batches

A solution of MB dye was prepared in deionized water with an initial concentration of 2000 mg L^−1^ and the initial pH of 7.0. About 21 mg of sorbent was added into 30 mL of MB solution in a polypropylene tube. Each sorbent along with a blank (without sorbent) were performed in 3 replications. The tubes were shaken at 150 rpm at 23 ± 2 °C and samples were drawn after 0, 2, 5, 15, 30, 60, 180, 300, 600, and 1440 min. The solution was separated from the sorbent and MB concentration was analyzed. The amount of the MB sorbed per unit mass of the sorbent was determined using Eq. (2).

Various kinetic models (Eq. 3–9) were used to investigate the sorption dynamics^23^.

3 $${\text{First-order}}\;\;\;\;lnq_{t} = lnq_{o} - k_{1} t$$

4 $${\text{Second-order}} \;\;\;\frac{1}{{q_{t} }} = \frac{1}{{q_{o} }} - k_{2} t$$

5 $${\text{Pseudo-first-order}}\;\;\;ln\left( {q_{e} - q_{t} } \right) = lnq_{e} - k^{\prime}_{1} t$$

6 $${\text{Pseudo-second-order}}\;\frac{t}{{q_{t} }} = \frac{1}{{k_{2}^{^{\prime}} q_{e}^{2} }} + \frac{1}{{q_{e} }}t$$

7 $${\text{Elovich}}\;\;q_{t} = \frac{1}{\beta }ln \left( {\alpha \beta } \right) + \frac{1}{\beta } lnt$$

8 $${\text{Power function}}\;\;\;lnq_{t} = lnb + k_{f} \left( {lnt} \right)$$

9 $${\text{Intraparticle diffusion}}\;\;\;q_{t} = c + k_{id} t^{0.5}$$

where *t* stands for time intervals, ${q}_{t}$ is the amounts of MB adsorbed at time *t* (mg g^−1^), and ${q}_{o}$ is the amounts of MB adsorbed time 0 (mg g^−1^). ${k}_{1}$ represents the first order rate constant, while ${k}_{2}$ is first- and second-order rate constant and ${q}_{e}$ stands for maximum sorption capacity (mg g^−1^). ${k}_{1}^{^{\prime}}$ stands for rate constant for pseudo-first order, and ${k}_{2}^{^{\prime}}$ stands for rate constant for pseudo-second-order, *α* represents the rate of initial sorption (mg g^−1^ min^−1^), while *β* is sorption constant, ${k}_{f}$ stands for rate coefficient (mg g^−1^ min^−1^), *b* represents rate constant, *c* stands for diffusion constant, and ${k}_{id}$ stands for apparent diffusion rate constant ([mg g^−1^]^−0.5^).

The closeness of modelling data with experimental data was assessed through calculating the standard error of estimate (*SEE*) as follows^24^:

10 $$SEE = \mathop \sum \limits_{i = 1}^{n} (q_{em} - q_{ec} )^{2}$$

where *n* stands for the number of measurements, ${q}_{em}$ is the measured and ${q}_{ec}$ is the calculated sorption capacities (mg g^−1^).

#### Equilibrium sorption batches

Different solutions with initial MB concentration of 0, 200, 400, 600, 800, 1000, 1500, and 2000 mg L^−1^, with initial solution pH of 7.0 were prepared in deionized water. Each sorbent material was suspended in 30 mL solution of MB at the rate of 0.70 g L^−1^. Each sorbent along with a blank (without sorbent) was performed in three repeats. The suspension was shaken for 24 h at 150 rpm at 23 ± 2 °C. Solution was separated from the sorbent materials and the concentration of MB in the solution was measured. Non-linear forms of different isotherm equations (Eqs. 11–15) were used to examine the sorption isotherms^25,26^.

11 $${\text{Langmuir}}\;\;\;q_{e} = \frac{{Q_{L} C_{e} K_{L} }}{{1 + K_{L} C_{e} }}$$

12 $${\text{Freundlich}}\;q_{e} = K_{F} C_{e}^{1/n}$$

13 $${\text{Temkin}}\;\;q_{e} = \frac{RT}{b}lnAC_{e}$$

14 $${\text{Dubinin-Radushkevich}}\;q_{e} = q_{D} {\exp}( - B_{D} \left[ {RTln \left( {1 + \frac{1}{{C_{e} }}} \right)]^{2} } \right)$$

15 $${\text{Redlich-Peterson}}\;q_{e} = \frac{{AC_{e} }}{{1 + { }BC_{e}^{g} }}$$

where, $Q_{L}$ represents the sorption capacity (mg g^−1^), and $K_{L}$ stands for sorption equilibrium constant (L mg^−1^). 1/*n* is the Freundlich component of linearity and $K_{F}$ stands for the Freundlich sorptive affinity (L g^−1^). *R* in Eq. (13) represents a universal gas constant with a constant value of 8.314 J K^−1^ mol^−1^, while *T* stands for absolute temperature, *A* stands for binding constant (L mg^−1^), and *b* represents the heat of adsorption (J mol^−1^). $q_{D}$ stands for maximum adsorption capacity (mg g^−1^), and *B*_*D*_ is the mean free energy of adsorption (kJ mol^−1^). In Eq. (15), *A* is the Redlich–Peterson constant in L g^−1^, and *B* is Redlich–Peterson constant in (L mg^−1^)^*g*^, while the value of the exponent (*g*) lies between zero and unity.

Estimated *B*_*D*_ from Eq. (14) was further used to calculate binding energy (*E*) as follows:

16 $$E = \frac{1}{{\sqrt {2B_{D} } }}$$

*Partition coefficient: *Partition coefficient (*K*_*P*_) is used to compare the sorptive capacities of different adsorbents for any specific adsorbate ion under same experimental conditions. It can be calculated by using Eq. (17)^27^.

17 $${K}_{P}=\frac{MB \, adsrobed \, at \, equilibrium}{MB \, concentration \, at \, equilibrium}$$

### Regeneration studies

The MB-loaded sorbents obtained from equilibrium sorption batches were separated from the solution and washed thrice with deionized water. Each of the washed sorbent (with three replications) was suspended in 30 mL of 0.1 M HCl solution and shaken for 600 min at 150 rpm at room temperature (23 ± 2 °C). Thereafter, the regenerated sorbent was separated from the solution, washed thrice with deionized water, dried in an over at 70 °C, and subjected to another cycle of MB sorption as discussed above (pH 7.0, contact time of 1440 min, initial MB concentration of 2000 mg L^−1^, and temperature 23 ± 2 °C). The above sorption–desorption cycle was repeated for four times to investigate the regeneration/reusability of each of the synthesized sorbent.

## Results and discussion

### Characterization

#### Proximate and chemical characteristics

The chemical and proximate characteristics of the produced sorbents are shown in Table 1. Pyrolysis of waste biomass resulted in an increment of 5.0 units in the pH of the biochar, which could be due to condensation of alkali-based functional groups, loss of acidic functional groups, and alkali salt separation^28^. However, reduction in pH in engineered biochar types could be due to washings of materials resulting in a subsequent loss of soluble basic cations^29^. The yield of DBC was found to be 31.78%. With pyrolysis, the volatile matter reduced significantly to 10.05% in DBC and 18.88% in nZVI-DBC, from 66.36% in DF, while it further increased to 73.69% in nCD-DBC. Unlike volatile material, the amount of fixed carbon increased to 50.10% in DBC compared with 2.50% in DF, while it reduced to 12.49% and 10.64% in nCD-DBC and nZVI-DBC, respectively.

**Table 1:** Proximate and chemical analyses result of date palm tree leaf waste biomass and different types of biochar derived from it.

	Yield		Moisture		Volatiles		Fixed carbon		Ash		pH		Cation exchangeable capacity	
	%		%		%		%		%		1:10		cmol kg^−1^	
DF	–		4.77 ± 0.03		66.36 ± 5.53		20.50 ± 5.72		8.36 ± 0.21		5.95 ± 0.00		66.01 ± 0.00	
DBC	31.78 ± 4.78		1.58 ± 0.75		10.05 ± 1.49		50.10 ± 0.98		38.27 ± 1.26		10.10 ± 0.02		57.83 ± 13.3	
nCD-DBC	–		1.32 ± 0.10		73.69 ± 0.21		12.49 ± 0.32		12.50 ± 0.02		7.42 ± 0.05		151.48 ± 5.16	
nZVI-DBC	–		3.96 ± 0.08		18.88 ± 1.23		10.64 ± 3.51		60.87 ± 7.31		6.15 ± 0.02		72.55 ± 3.95	

*DF* date palm waste biomass, *DBC* date palm waste-derived biochar, *nCD-DBC* date palm waste-derived carbon nanodots, *nZVI-DBC* date palm waste-derived biochar composite with nano zero-valent iron.

Reduction in the resident matter in engineered biochars could be due to the presence of indecomposable substances that were muddled with ash contents. The contents of ash significantly increased from 8.36% in DF to 38.27% in DBC as a result of condensation and production of compounds of minerals during the thermalization process^30^. Interestingly, ash contents were reduced in nCD-DBC (12.5%), which may be attributed to several washings, while it increased further to 60.87% in nZVI-DBC due to the occurrence of iron compounds, such as graphene and Fe_3_C. The point of zero charges (PZC) for the produced sorbents are presented in Fig. S1 (Supplementary data). The PZC is the pH at which the sum of anions and cations is at equilibrium. Results revealed that the PZC for DBC, nCD-DBC and nZVI-DBC was 8.71, 7.82, and 7.22, respectively.

#### Distribution of particle size

The size of the particles in biochar is highly dependent on pyrolytic conditions and the nature of the feedstock used. The particle size of engineered/designer biochar depends on mechanical, chemical, or physical treatment of the materials. The particle size distribution of the produced adsorbents has been shown in Fig. S2. The results revealed that the particles with size of 200–800 μm were maximum in DBC sample, while a larger proportion of the particles was of size 5.0–100 μm in nZVI-DBC sample. The particle size of nCD-DBC was very small, where 35.95% of the particles were in size range of 5.0–10 nm, and a larger proportion of the particles was found in size range of 5.0–20 nm (Fig. S2c).

#### SEM, XRD, FTIR, and TGA analyses

**Figure 1:**
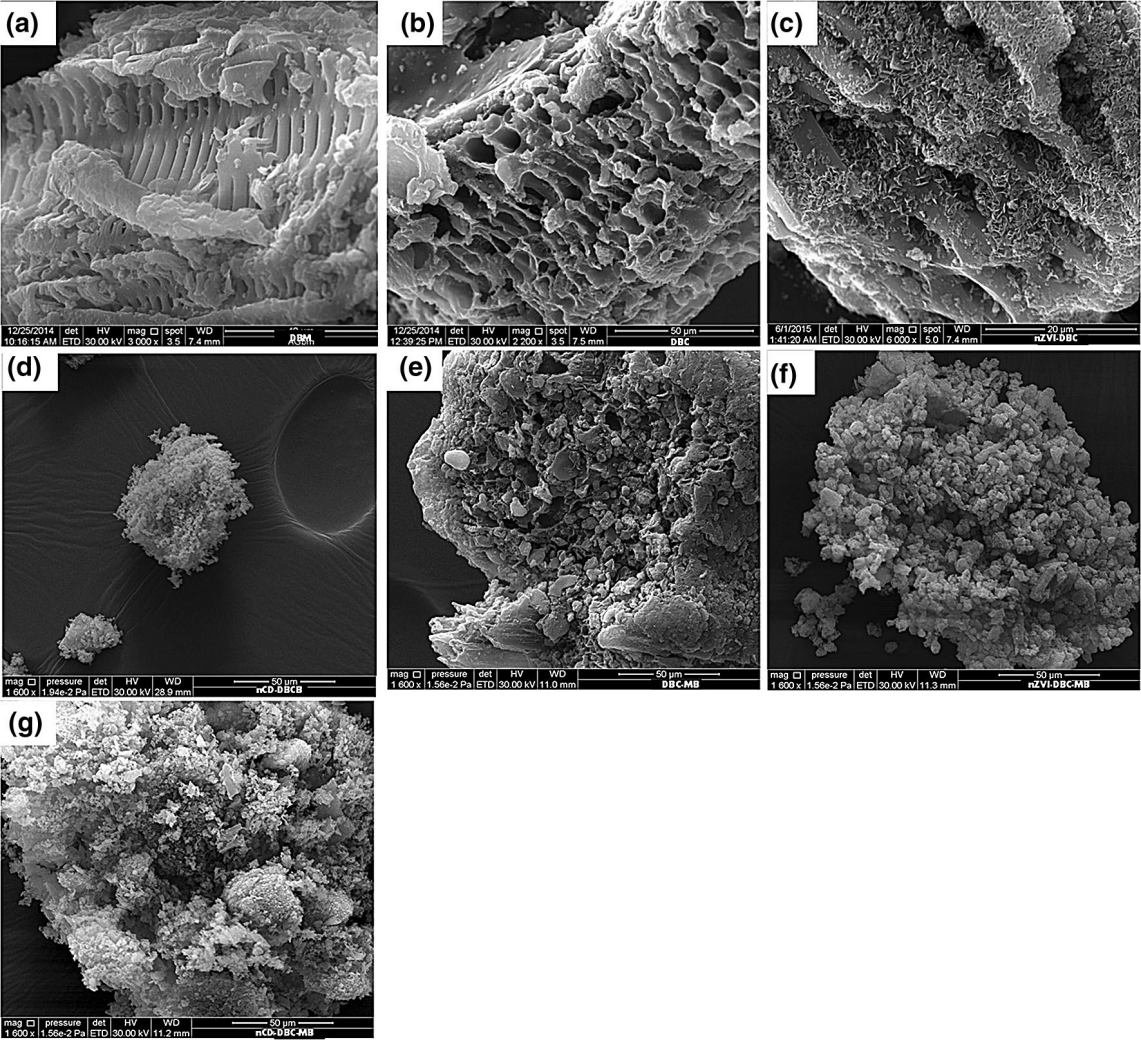
Scanning electron microscope (SEM) images of (**a**) date palm waste feedstock (DF), (**b**) date palm waste derived biochar (DBC), (**c**) date palm waste derived biochar composite with nano zerovalent iron (nZVI-DBC), (**d**) nano carbon dots (nCD-DBC), (**e**) date palm waste derived biochar loaded with methylthioninium chloride (DBC-MB), (**f**) date palm waste derived biochar composite with nano zerovalent iron loaded with methylthioninium chloride (nZVI-DBC-MB), and (**g**) nano carbon dots loaded with methylthioninium chloride (nCD-DBC).

**Figure 2:**
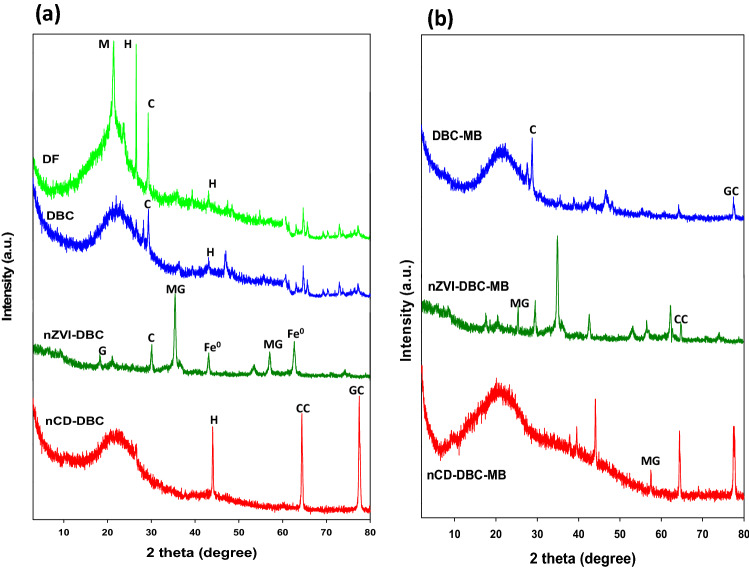
XRD (X-ray diffraction) patterns of date palm waste feedstock (DF), date palm waste derived biochar (DBC), date palm waste derived carbon nanodots (nCD-DBC), and date palm waste derived biochar composited with nano zerovalent iron (nZVI-DBC) before (**a**) and after (**b**) the sorption of methylthioninium chloride (MB) from aqueous media. *H* halite, *M* mellite, *C* calcite, *G* graphene, *Fe*^*0*^ nano zerovalent iron, *MG* magnetite, *CC* turbostratic carbon, *GC* graphite-like carbon nanofibers.

**Figure 3:**
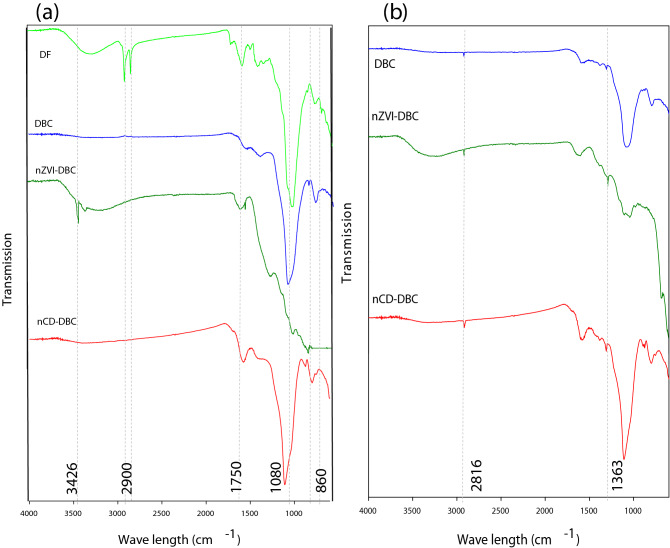
FTIR (Fourier transform infrared spectroscopy) spectra of date palm waste derived biochar (DBC), date palm waste derived carbon nanodots (nCD-DBC) and date palm waste derived biochar composited with nano zerovalent iron (nZVI-DBC-). (**a**) before sorption of methylthioninium chloride, and (**b**) after the sorption of methylthioninium chloride.

The morphology of the sorbents as assessed through SEM images is shown in Fig. 1. It was observed that the surface of the DF was crystalline and smooth (Fig. 1a), which turned into porous and non-crystalline after thermal treatment (Fig. 1b). Additionally, the presence of channels on the surface of DBC indicated the loss of volatiles components during pyrolysis. The existence of tiny rods on the surface of nZVI-DBC suggested the formation of Fe^0^ particles on the biochar matrix (Fig. 1c). Figure 1d showed that nCD-DBC particles were very small in size, highly amorphous, and agglomerated that resulted in the formation of bigger particles. The XRD patterns of synthesized sorbents are shown in Fig. 2a. Various peaks representing different mineralogical phases were detected in the XRD patterns. A high-intensity peak at 21.48° in DF was attributed to mellite; however, it vanished after pyrolysis in all other sorbents. Likewise, a peak at 28°, which was designated to originate from a halite, was visible in DF and disappeared in all other sorbents. Another peak representing halite appeared at 43° with lower intensity in DF and DBC and with higher intensity in nCD-DBC. A peak at 29.3° in DF was attributed to calcite, which was reduced in DBC and nZVI-DBC, whereas it completely vanished in nCD-DBC. Interestingly, the synthesis of graphene was also witnessed with the appearance of a peak at 18.5° in nZVI-DBC. Two peaks appearing at 44° and 63° in nZVI-DBC were ascribed as Fe^0^ nanoparticles, confirming the synthesis of nZVI particles during composite formation^13^. The peaks appearing at 35° and 55° in nZVI-DBC were designated as magnetite. The XRD patterns of nCD-DBC were distinguished from other sorbents with the appearance of two intense peaks at 64° and 77.5°, providing evidence of the synthesis of carbon nanodots. The peak appearing at 64° in nCD-DBC was attributed to crystalline carbon (turbostratic carbon), while a peak at 77.5° was speculated to originate from graphite-like carbon nanofibers. The FTIR spectra of the pristine biomass and derived sorbents are shown in Fig. 3a. A wide band around 3300 cm^−1^ in samples of nZVI-DBC and DF ascribed as O–H stretching of H_2_O molecules, which were reduced to a minimal level in DBC and nCD-DBC, suggesting lower moisture contents in these materials. These results can be compared with the moisture contents presented in Table 1, suggesting higher moisture contents in DF and nZVI-DBC (4.77% and 3.96%, respectively), while lower moisture contents in DBC and nCD-DBC (1.58% and 1.32%, respectively). A band appearing at 3426 cm^−1^ in the composite of nZVI-DBC was attributed to –OH stretching vibrations, which indicated the presence of a layer of FeOOH onto the surfaces of nZVI particles. A peak appearing at 2900 cm^−1^ in DF was designated to aliphatic C–H and O–H stretching owing to cellulose and hemicellulose, which was vanished in DBC^31^. A band around 1750 cm^−1^ in DF represented the existence of C=O stretching. The vanishing of the band around 1750 cm^−1^ in DBC suggested the degradation of hemicellulosic compounds during pyrolysis. This band further appeared in nZVI-DBC and nCD-DBC due to overlapping with N–H groups of the chitosan. A band around 1080 cm^−1^ was credited to C–O–C stretching vibrations suggesting the presence of undecomposed oxygenated groups on the organic compounds in biochar^32,33^. Few smaller peaks around 860 cm^−1^ were attributed to the presence of C–O stretching. The thermograms of the sorbents as obtained from TGA analyses are presented in Fig. S3a. First weight loss occurred around 200 °C in all the materials owing to the removal of water from the surface and pores of the materials. A sudden weight loss was observed around 300 °C in DF, as a result of degradation of cellulose and hemicellulose based compounds during thermalization. Likewise, a sudden weight loss as a result of decomposition of cellulosic and hemicellulosic compounds occurred around 400, 450, 500 °C in DBC, nCD-DBC, and nZVI-DBC, respectively. The maximum weight loss was in the following order: DF (81.14%) > nCD-DBC (41.01%) > DBC (62.63%) > nZVI-DBC (41.23%). Thermal stability of the materials was further estimated by calculating the recalcitrance potential (*R*_*50*_) of ash and moisture-free TGA thermograms as reported by Ahmad et al.^31^. It has been reported that materials with *R*_*50*_ < 0.50 are highly degradable, 0.70 > *R*_*50*_ ≥ 0.50 are marginally stable, and those with *R*_*50*_ > 0.70 are highly stable^31^. As presented in Fig. S3b, the thermal stability of the studied materials was in the order of: nCD-DBC (0.71) > DBC (0.62) > nZVI-DBC (0.59) > DF (0.44). Hence, it can be observed that DF was highly degradable, DBC and nZVI-DBC were marginally stable, while nCD-DBC was highly stable.

### MB sorption batches

#### Effects of pH on MB sorption

**Figure 4:**
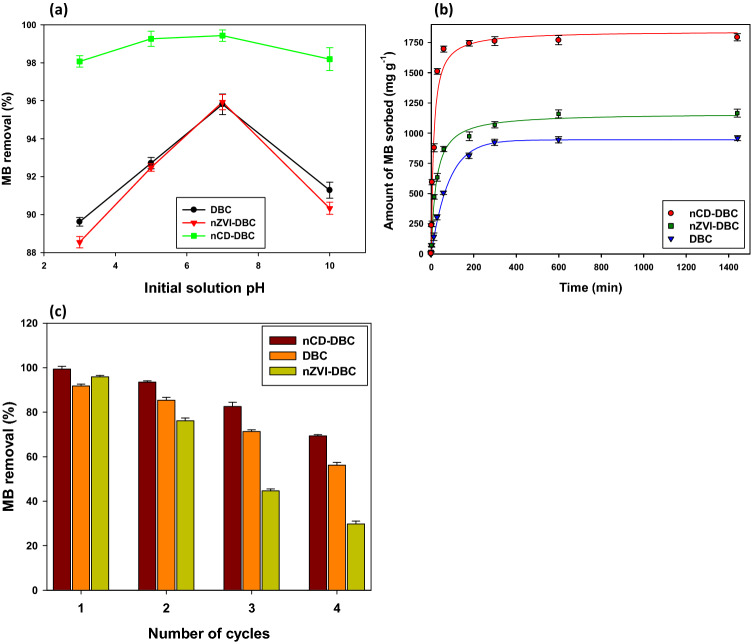
Effect of initial solution pH on methylthioninium chloride (MB) removal efficiency (**a**), effect of contact time on MB adsorption (**b**), and regeneration cycles for the adsorption of MB (**c**) onto date palm waste derived biochar (DBC), date palm waste derived carbon nanodots (nCD-DBC), and date palm waste derived biochar composited with nano zerovalent iron (nZVI-DBC).

The removal efficiency of sorbents largely relies on surface and structural characteristics of adsorbate and adsorbent, which in turn, are influenced by the pH. Therefore, any change in solution pH, changes the composition of charges on the surface of both adsorbate and adsorbent, and thus, influences the properties of materials, including their stability, ion exchange capacity, interaction with electrolytes, and suspension rheology. The effects of pH on the removal of MB dye through synthesized sorbents were investigated in the initial solution pH range of 3.0–10 and the results are presented in Fig. 4a. It was noticed that the removal percentage increased with an increase in solution pH from 3.0 to 7.0 and decreased thereafter. The maximum removal percentage occurred at neutral pH. Interestingly, nCD-DBC removed a higher amount of MB dye at all the pH ranges as compared to other sorbents. A competitive sorption between H^+^ ions and MB dye (cationic dye) at lower pH resulted in lower sorption efficiency due to electrostatic repulsive interactions^34^. An increase in pH from 3.0 to 7.0 resulted in higher OH^–^ ion concentrations, consequently resulting in enhanced negative charges on the surfaces. The anionic charges dominated the surface at pH 7.0, which resulted in cation exchange reactions, and thus, higher sorption efficiencies^35^. The sharp downfall in the removal efficiency after pH 7.0 could be owing to surface charge balancing as indicated by the PZC values. Hence, at a lower pH, the surfaces of the sorbents were dominated by positive charges, which tended to repel the positively charged MB dye ions resulting in lower removal efficiency^36^. However, as the pH of the solution increased from 3.0 to 7.0, the concentration of negative charges was increased on the surface of the sorbents, which in turn resulted in a higher removal efficiency at pH 7.0.

#### Kinetic sorption

Kinetic sorption batches were performed to investigate the dynamics of MB sorption onto the synthesized sorbents at a constant temperature under optimal pH conditions (Fig. 4b). Generally, three sorption phases were identified in the MB sorption dynamics, including a rapid (0–100 min), slow (100–300 min), and equilibrium (300–1440 min) phase. The higher sorption efficiencies of the sorbents at the beginning were due to higher availability of the sorption sites, which were then occupied, resulting in slow sorption until equilibrium was gradually reached. The nCD-DBC exhibited a higher rate of reaction and sorption capacity, followed by nZVI-DBC and DBC. The estimated error functions (*SEE*) and correlation coefficient (*R*^*2*^) are shown in Table 2 to explain the suitability of the kinetic models for experimental sorption data. The estimated *R*^*2*^ values suggested that pseudo-second-order (0.93–0.99) and Elovich models (0.86–0.97) were well fitted; while second-order (0.74–0.87), power function (0.51–0.81), and intraparticle diffusion (0.50–0.76) were slightly fit, and first-order (0.15–0.23) and pseudo-first-order (0.07–0.08) were unfit to explain the sorption kinetics. The *SEE* for all the sorbents in all kinetic models was less than 1.0 suggesting lower error possibilities. The parameters calculated from the kinetic modeling are shown in Table 2. Second-order, power function, and pseudo-second-order predicted rate constants were highest for nCD-DBC (*k*^*2*^ = –6.8 × 10^–7^, *b* = 3.821, and *k*^2*′*^ = –7.1 × 10^–5^, respectively). As the rate constants define the time taken by a chemical process to be completed, therefore, higher rate constants for nCD-DBC suggested its higher affinity for MB sorption than other sorbents. The best fitness of Elovich and pseudo-second-order models indicated that the sorption of MB was being controlled by chemisorption. Likewise, nCD-DBC exhibited highest values of *h* and *k*_*id*_ (227.50 mg g^−1^ min^−1^ and 41.25 [mg g^−1^]^−0.5^, respectively) than the other tested sorbents. Similarly, the diffusion constant (*c*) was maximum for nCD-DBC (724.82), followed by nZVI-DBC (311.92), and DBC (140.11). Therefore, it can be stated that MB removal was also aided by intraparticle diffusion process. The highest apparent diffusion rate and diffusion constant of nCD-DBC suggested a quick diffusion of MB cations onto the said sorbent.

**Table 2:** Coefficients of determination (*R*^*2*^), standard errors of estimate (*SEE*) and other parameters obtained from kinetic models for methylthioninium chloride sorption onto date palm waste-derived biochar (DBC), date palm waste-derived carbon nanodots (nCD-DBC), and date palm waste-derived biochar composited with nano zero-valent iron (nZVI-DBC).

Sorbent	Parameter	nCD-DBC	nZVI-DBC	DBC
First-order	*k*_*1*_	1.6 × 10^–3^	1.8 × 10^–3^	2.3 × 10^–3^
	*R*^*2*^	0.15	0.10	0.23
	*SEE*	0.002	0.009	0.031
Second-order	*k*_*2*_	− 6.8 × 10^–7^	− 2.3 × 10^–6^	− 1.2 × 10^–5^
	*R*^*2*^	0.74	0.87	0.79
	*SEE*	0.009	0.020	0.899
Pseudo-first-order	*k*_*1*_^*'*^	− 4.8 × 10^–3^	− 5.1 × 10^–3^	− 5.4 × 10^–3^
	*q*_*e*_	5.99	6.38	6.23
	*R*^*2*^	0.07	0.08	0.07
	*SEE*	0.025	0.019	0.030
Pseudo-second-order	*k*_*2*_^*'*^	7.1 × 10^–5^	3.7 × 10^–5^	1.7 × 10^–5^
	*q*_*e*_	1793.40	1182.80	1008.80
	*h*	227.50	52.22	17.21
	*R*^*2*^	0.93	0.99	0.99
	*SEE*	0.001	0.001	0.001
Elovich	*a*	179.77	267.71	161.17
	*β*	202.23	− 3.11	− 122.28
	*R*^*2*^	0.86	0.97	0.94
	*SEE*	0.046	0.062	0.023
Intraparticle diffusion	*k*_*id*_	41.25	30.85	29.41
	*c*	724.82	311.92	140.11
	*R*^*2*^	0.50	0.70	0.76
	*SEE*	0.1137	0.0116	0.0133
Power function	*k*_*f*_	0.68	0.71	0.83
	*b*	3.82	3.11	2.07
	*R*^*2*^	0.51	0.62	0.81
	*SEE*	0.001	0.000	0.000

#### Equilibrium sorption

**Figure 5:**
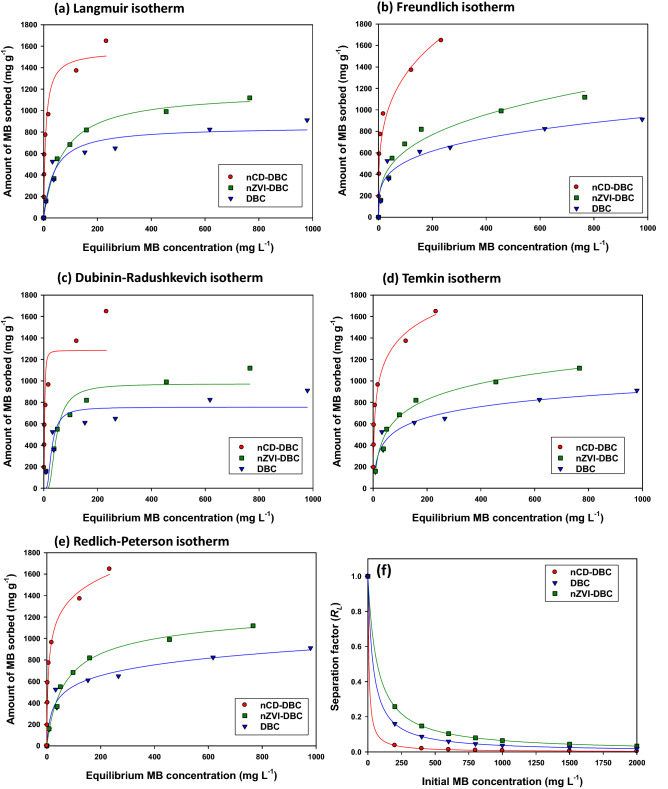
Methylthioninium chloride (MB) sorption isotherms fitting on Langmuir (**a**), Freundlich (**b**), Debinin-Radushkevich (**c**), Temkin (**d**), and Redlich-Peterson models (**e**), along with separation factor (*R*_*L*_) (**f**) for date palm waste derived biochar (DBC), date palm waste derived carbon nanodots (nCD-DBC) and date palm waste derived biochar composited with nano zerovalent iron (nZVI-DBC).

Sorption isotherm studies describe the principle behind the mobility or retention of an adsorbate from aqueous solution to an adsorbent at constant temperature in the form of special curves^37^. The accuracy of isotherm models can be explained by the function of independent parameters, whereas, its mathematical simplicity is indicated by its popularity concerning the process application^24^. Based on the results of the pH batches, the isotherm sorption batches were performed at an initial solution pH of 7.0. To describe and understand the sorption of MB onto nCD-DBC, nZVI-DBC, and DBC, non-linear forms of Freundlich, Langmuir, Dubinin-Radushkevich, Temkin, and Redlich-Peterson models were fitted to the experimental sorption data (Fig. 5a–e, respectively). The amount of MB sorbed onto the synthesized sorbents increased with an increase in the initial concentration of ions in the solution^38^. All the isotherms exhibited three phases of sorption, i.e., an H-type (high affinity) isotherm at lower initial MB concentrations, followed by an L-type (low affinity) isotherm at higher initial MB concentrations until an equilibrium sorption phase was achieved. The higher MB sorption at lower initial concentration could be owing to the presence of more active sites, which were reduced subsequently with a rise in the initial MB concentration in the solution^13^. Non-linear parameters as acquired from isotherm modelling data are provided in Table 3. The fitness of the isotherm models to describe MB sorption onto the synthesized sorbents was in the order: Redlich-Peterson > Langmuir ≥ Temkin > Freundlich > Dubinin-Radushkevich. Langmuir isotherm predicted *Q*_*L*_ was highest for nCD-DBC (1558.66 mg g^−^1), while nZVI-DBC and DBC shown comparatively lower *Q*_*L*_ values (1182.90, and 851.67 mg g^−1^, respectively). The best fitness of Langmuir model suggested monolayer sorption of MB dye onto the surface of the tested sorbents. The sorptive affinity, as predicted by Freundlich isotherm, followed a similar trend as: nCD-DBC (378.00 L g^−1^) > nZVI-DBC (156.67 L g^−1^) > DBC (144.65 L g^−1^), indicating the highest sorption of MB onto nCD-DBC. As the Freundlich model is an empirical relationship between the concentration of sorbate and its heterogeneous surface, hence, it is assumed that the sorbate first occupies the stronger binding sites; afterward, the strength of binding decreases with an increase in the degree of occupation^24^. Furthermore, the sorption of MB on nCD-DBC, nZVI-DBC, and DBC fitted well into the Redlich-Peterson isotherm with *R*^2^ of 0.98, 0.99, and 0.96, respectively, suggesting the involvement of multiple sorption mechanisms^39^. The Redlich-Peterson isotherm assimilates the features of both Freundlich and Langmuir isotherms; thus, it is suitable for application on both heterogeneous and homogenous systems. The values of *g* as predicted by Redlich–Peterson model were lower than unity in all the sorbents, suggesting a higher fitness of the Langmuir isotherm than the Freundlich isotherm. Temkin isotherm predicted *b* was the highest in nCD-DBC (263.70 J mol^−1^). Likewise, Dubinin-Radushkevich isotherm predicted *Q*_*D*_ was also the highest in nCD-DBC (1283.40 mg g^−1^). The calculated binding energy (*E*) for all the sorbents was very low (0.01, 0.27, and 0.66 kJ g^−1^ for nCD-DBC, nZVI-DBC, and DBC, respectively), indicating that MB sorption onto the sorbents did not follow the ion-exchange process^23^. The favorability of sorption was further assessed through the 1*/n* value predicted from the Freundlich isotherm. It has been established that a value of 1*/n* < 0.50 implies a highly favorable sorption, while a value of 1*/n* > 1.0 implies an unfavorable sorption process^23^. In this study, the values of 1*/n* were 0.27, 0.26, and 0.32 for nCD-DBC, nZVI-DBC, and DBC, respectively, indicating a higher sorption affinity of the synthesized sorbents for MB. Additionally, the separation factor (*R*_*L*_) for all sorbents was less than 1.0, suggesting favorability of the MB sorption onto the sorbents (Fig. 5f). Furthermore, the nCD-DBC exhibited a higher favorability of MB sorption by its lower *R*_*L*_ value as compared to nZVI-DBC and DBC. The highest sorptive affinity of nCD-DBC towards MB dye was also evidenced by its highest *K*_*P*_ value (7067 mL g−^−1^), compared with nZVI-DBC (1460 mL g^−1^) and DBC (930 mL g^−1^) (Table 3). *K*_*P*_ is considered as a crucial parameter to predict the sorptive performance of a particular adsorbent and generally varies with particle size and PZC of adsorbent as well as the pH of the solution^40^. Thus, *K*_*P*_ minimizes the bias of concentration effect and predicts the real performance of the adsorbents. Therefore, the highest *K*_*P*_ value for nCD-DBC can be designated to its stronger sorptive affinity towards MB than other tested adsorbents^41^. Larger *K*_*P*_ values at higher adsorption capacities have already been reported^42^. Hence, these results suggested that the synthesized sorbents effectively sorbed the MB dye from aqueous media through the mono– and multi-layer sorption process. Moreover, nCD-DBC performed better by exhibiting the highest MB sorption capacity as compared to nZVI-DBC and DBC. Maximum adsorption capacities of various adsorbents towards MB dye investigated previously in comparison with the adsorption efficiencies of the adsorbents tested in this study have been listed in Table S1. It is obvious from the presented data that the maximum MB adsorption capacity of all the adsorbents was lower than nCD-DBC. However, the maximum adsorption capacities of calcium alginate beads composites with bamboo biochar, eucalyptus residue-activated carbon with H_3_PO_4_, and NaOH-activated carbon were higher (1210.70–1282.20 mg g^−1^, 980.39–1000 mg g^−1^, and 916.26 mg g^−1^, respectively) compared with nZVI-DBC and DBC^43–45^.

**Table 3:** Non-linear parameters of Langmuir, Freundlich, Redlich-Peterson, Temkin, and Dubinin–Radushkevich isotherms, along with partition coefficient for methylthioninium chloride sorption onto date palm waste-derived biochar (DBC), date palm waste-derived carbon nanodots (nCD-DBC), and date palm waste-derived biochar composited with nano zero-valent iron (nZVI-DBC).

Isotherms	Parameters		nCD-DBC	nZVI-DBC	DBC
Langmuir	*Q*_*L*_	(mg g− ^1^)	1558.66	1182.90	851.67
	*K*_*L*_	(L g− ^1^)	0.13	0.02	0.03
	*R*^*2*^		0.96	0.99	0.94
Freundlich	*K*_*F*_	(L g− ^1^)	378.00	156.67	144.65
	*1/n*		0.27	0.26	0.32
	*R*^*2*^		0.95	0.96	0.95
Redlich-Peterson	*A*	(L g− ^1^)	282.59	39.79	19.29
	*B*	(L mg− ^1^)^g^	0.33	0.10	0.02
	*g*		0.86	0.92	0.82
	*R*^*2*^		0.98	0.99	0.96
Temkin	*b*	(J mol^−1^)	263.70	217.86	146.83
	*A*	(L g^−1^)	1.99	0.44	0. 22
	*R*^*2*^		0.97	0.98	0.94
Dubinin–Radushkevich	*Q*_*D*_	(mg g^−1^)	1283.40	972.97	755.04
	*E*	(kJ g^−1^)	0.01	0.27	0.66
	*R*^*2*^		0.81	0.89	0.75
Partition coefficient	*K*_*P*_ (mL g^−1^)		7067	1460	930

### Recyclability of synthesized sorbents

The reusability of the synthesized sorbents has been investigated using four cycles of regeneration to access the practical application (Fig. 4c). The MB removal efficiency of the sorbents reduced substantially after the first regeneration cycle. It was noticed that the MB removal efficiency of nCD-DBC was reduced to 69.31% after 4 cycles of regeneration which is quite higher. However, the MB removal efficiency of DBC and nZVI-DBC reduced to 56.22% and 29.78%, respectively, after 4 cycles of regeneration. The decreasing tendency of the sorbents for MB sorption with more regeneration cycles could be owing to reduced surface area and diminishing of the polar functional groups of the sorbents^46^. Moreover, the lowest reusability of the nZVI-DBC than the other tested sorbents in this study could be owing to the dissociation and oxidation of Fe^0^ particles^47^. The higher regeneration capability of the nCD-DBC depicted its potential applicability to decontaminate the MB-containing wastewater.

### Mechanisms for MB removal

The sorption of MB onto the sorbents was lower at acidic pH due to the abundance of H^+^ on the surface of aromatic sorbents, which likely created repulsive forces between H^+^ charges on sorbents and the cationic MB dye. With an increase in pH, the concentration of H^+^ reduced, consequently increasing the negative charges, resulting in higher MB sorption. However, as pH crossed the PZC, the sorption of MB dropped sharply due to charge balancing on the surface of the sorbents. Thus, it can be stated that the electrostatic interactions were involved in MB sorption onto the studied sorbents^48^. Furthermore, the occurrence of various functional groups on the surfaces of the sorbents (Fig. 3b) may result in generation of some negative charges, consequently aiding the MB removal process. For instance, the hydrogen bond that exists between the –OH groups of the sorbents and the –N-groups of MB could contribute to the removal of MB dye as well^49^. Thus, electrostatic interactions between cationic MB and anionic surface functional groups were the major MB removal mechanisms.

The presence of more active sites and highest surface areas of nZVI-DBC and nCD-DBC significantly augmented the MB sorption process as compared to DBC. The highest MB removal through nCD-DBC could be designated to its nano sized structure (5.0–20 nm; Fig. S2) with enhanced surface interactions between the sorbate and sorbents. The highest surface area helped in MB removal through π-π electron donor-accepter interactions between the aromatic skeletons of the tested sorbents and MB cations^50^. Further, the highest MB removal efficiency of nCD-DBC as compared to the other tested sorbents could be owing to the instantaneous availability of the active sorption sites on the surface allowing a rapid diffusion within the matrix as is obvious from the highest apparent diffusion rate and diffusion constant. However, the kinetics sorption data revealed that ion-exchange was not involved in MB sorption onto any of the studied sorbents (also obvious from lower *E* values (< 8.00 kJ g^−1^) for all the tested sorbents in Table 3). The best fitness of Elovich and pseudo-second-order models indicated the involvement of chemisorption in the sorption of MB onto the sorbents.

Moreover, the nZVI particles could effectively degrade the MB molecules via redox-reactions. nZVI particles act as electron donor and upon dissolution, an Fe^0^ nanoparticle emits 2e^–^ by oxidizing itself (as shown in Eq. 18). The emitted electrons are transferred into MB dye molecule as it acts as electron acceptor, consequently reducing it into colorless leucomethylene^51^.

18 $${\text{Fe}}^{0} + {\text{ O}}_{{2}} + {\text{ 2H}}^{ + } \to {\text{ Fe}}^{{{2} + }} + {\text{ H}}_{{2}} {\text{O}}_{{2}}$$

Further, the reaction of Fe^2+^ with H_2_O_2_ results in the formation of hydroxyl radicals (•OH) (Eq. 19) with strong oxidizing capability.

19 $${\text{Fe}}^{{{2} + }} + {\text{ H}}_{{2}} {\text{O}}_{{2}} \to {\text{ Fe}}^{{{2} + }} + \, \cdot {\text{OH }} + {\text{ OH}}^{-}$$

The produced •OH radicals results in the degradation of MB dye as mentioned in Eq. (20).

20 $${\text{MB}} + \cdot {\text{OH}} \to {\text{Intermediates }} \to \, \cdot {\text{OH }} \to {\text{ CO}}_{{2}} + {\text{ H}}_{{2}} {\text{O }} + {\text{ mineral products}}$$

However, as the pH exceeded 6.0, the Fe^2+^ was precipitated as iron oxides/hydroxides, which occupied the surface of nZVI-DBC matrix, subsequently blocking the transfer of electrons from Fe^0^ to MB; hence, reducing the removal of MB^52^. Furthermore, besides electrostatic interactions, redox reactions, reduction, and π–π electron donor-accepter interactions were also involved for MB removal through nZVI-DBC.

It has been reported earlier that carbon dots can act both as electron donor and acceptor^53^. This capability of nCD-DBC might have resulted in excellent removal of MB dye from aqueous solutions due to π-π electron donor-accepter interactions. Additionally, few researchers have reported that carbon dots can generate as well as scavenge the free radicals in aqueous solutions^54,55^. The oxygen free radicals (O_2_•^−^) generated by MB dye, due to its oxygen sensitizer property might have been scavenged by the nCD-DBC in aqueous solution, whereas, the •OH free radicals generated by the nCD-DBC successfully degraded the MB dye. Therefore, π–π electron donor–accepter interactions and degradation due to free radicals were the principal MB removal mechanisms by nCD-DBC.

The sorption of MB onto the sorbents was further confirmed by FTIR spectra, XRD patterns, and SEM images of the MB-loaded sorbents. The appearance of two new peaks at 1363 and 2816 cm^−1^ in FTIR spectra were attributed to MB dye (Fig. 3b), indicating the sorption of MB onto the surface of the sorbents. Further, a band at 3426 cm^−1^, representing FeOOH layer onto nZVI particles, disappeared. Likewise, changes in peak intensities and appearance of new peaks in XRD patterns of MB-loaded sorbents were obvious (Fig. 2b). The intensities of peaks representing turbostratic carbon (64°), graphite-like carbon (77.5°), and Fe^0^ particles (63°) decreased, whereas, the intensities of peaks representing magnetite (35°) increased with MB sorption onto the nCD-DBC and nZVI-DBC. Contrarily, the intensity of graphite-like carbon peak at 77° in DBC increased with MB sorption. A new peak appearing around 65° in nZVI-DBC was designated to turbostratic carbon, while a new peak appearing at 57° in nCD-DBC was ascribed as magnetite after MB sorption. Post-sorption SEM images (Fig. 1e–g) represented the changes in the morphology of the sorbents and the presence of some particles on the surface indicating the sorption of MB. Hence, these analyses depicted the interactions of adsorbate with the sorbents, subsequently adsorbing and removing the MB from water. Further, the simulations with kinetic and isotherm modelling and post-sorption analyses suggested electrostatic interaction, π–π electron donor-accepter interaction, diffusion, and degradation as the possible mechanisms for the removal of MB dye through DBC, nCD-DBC, and nZVI-DBC.

## Conclusions

The synthesized date palm waste derived sorbents were found very efficient for methylthioninium chloride (MB) removal from aqueous solutions. The maximum MB sorption was achieved at a solution pH of 7.0. The nCD-DBC exhibited a higher rate of reaction and sorption capacity as compared to the other tested sorbents. Furthermore, the sorption kinetics of MB dye onto DBC, nCD-DBC, and nZVI-DBC were best fitted to pseudo-second-order and Elovich models suggesting that the sorbents followed chemisorption. Additionally, the diffusion was involved in the process of sorption, whereas, nCD-DBC showed the highest apparent diffusion rate and diffusion constant. Concerning the adsorption isotherm studies, the performance of nCD-DBC surpassed other sorbents and exhibited the highest sorption capacity (1558.66 mg g^−1^). The same trend was observed by the Dubinin-Radushkevich, Freundlich, Redlich-Peterson, and Temkin isotherms. Electrostatic interactions due to anionic surface functional groups, π–π electron donor-accepter interactions due to aromatic structure of adsorbate and adsorbent, and degradation due to free hydroxyl radicals were the major mechanisms controlling MB removal. Overall, nCD-DBC and nZVI-DBC showed 83% and 38% higher MB removal, respectively, than pristine biochar, suggesting that nCD-DBC and nZVI-DBC could potentially be employed as efficient, environment friendly, and cost-effective sorbents for MB removal from contaminated water.

## Supplementary information

Supplementary file1
