# Supplementary material for: Turning date palm waste into carbon nanodots and nano zerovalent iron composites for excellent removal of methylthioninium chloride from water
Source: Sci Rep. 2020 Sep 30;10:16125. doi: 10.1038/s41598-020-73097-x (PMC7527963; doi:10.1038/s41598-020-73097-x)
Supplement: Supplementary file 1 — Supplementary file1 [file 41598_2020_73097_MOESM1_ESM.docx]

**Supplementary data**

**Turning date palm waste into carbon nanodots and nano zerovalent iron composites for excellent removal of methylthioninium chloride from water**

Munir Ahmad^a^, Mutair A. Akanji^a^, Adel R.A. Usman^a,b^, Abdullah S.F. Al-Farraj^a^, Yiu Fai Tsang^c^, Mohammad I. Al-Wabel^a,c^*

^a^Soil Sciences Department, College of Food & Agricultural Sciences, King Saud University, P.O.

Box 2460, Riyadh 11451, Kingdom of Saudi Arabia

^b^Department of Soils and Water, Faculty of Agriculture, Assiut University, Assiut 71526, Egypt

^c^Department of Science and Environmental Studies, The Education University of Hong Kong,

Hong Kong

Corresponding author

Email: [malwabel@ksu.edu.sa](mailto:malwabel@ksu.edu.sa)

Phone: +966-1-467-8441

Fax: +966-1-467-8440

**Table S1.** Comparison of maximum adsorption capacity of various adsorbents as predicted by Langmuir isotherm for methylthioninium chloride sorption (date palm waste-derived biochar (DBC), date palm waste-derived carbon nanodots (nCD-DBC), and date palm waste-derived biochar composited with nano zero-valent iron (nZVI-DBC)).

| **Order** | **Sorbent** | **Adsorption temperature (°C)** | **Adsorption pH** | **Equilibrium time (min)** | **Langmuir isotherm predicted maximum adsorption capacity (mg g^-1^)** | **Reference** |
| --- | --- | --- | --- | --- | --- | --- |
| 1 | nCD-DBC | 23 | 7 | 1440 | 1558.6 | This study |
| 2 | nZVI-DBC | 23 | 7 | 1440 | 1182.9 |  |
| 3 | DBC | 23 | 7 | 1440 | 851.67 |  |
| 4 | Calcium alginate beads | 25 | 5 | 1440 | 1282.20 | Wang et al.^1^ |
| 5 | Ball milled bamboo biochar+ calcium alginate beads | 25 | 5 | 1440 | 1210.70 |  |
| 6 | Eucalyptus residue-activated carbon+H_3_PO_4_ | 25 | 7 | 1440 | 1000 | Han et al.^2^ |
| 7 | Eucalyptus residue-activated carbon+H_3_PO_4_ | 35 | 7 | 1440 | 990.10 |  |
| 8 | Eucalyptus residue-activated carbon+H_3_PO_4_ | 45 | 7 | 1440 | 980.39 |  |
| 9 | NaOH-activated carbon | 25 | 6.5 | 150 | 916.26 | Cazetta et al.^3^ |
| 10 | Thiosemicarbazide functionalized graphene oxide | 30 | -- | 120 | 724.6377 | Bu et al.^4^ |
| 11 | Core@double-shell structured HNTs/Fe_3_O_4_/ poly(DA + KH550) nano-hybrids | 25 | 10 | 180 | 714.29 | Wan et al.^5^ |
| 12 | Metal-organic framework UiO-66 | 25 | 10 | 300 | 543.48 | Song et al.^6^ |
| 13 | Bamboo-based activated carbon+KOH | 30 | 7 | 2880 | 454.2 | Hameed et al.^7^ |
| 14 | Graphene oxide modified persimmon tannin | 30 | 8 | 180 | 444.44 | Wang et al.^8^ |
| 15 | Graphene oxide modified persimmon tannin | 40 | 8 | 180 | 434.38 |  |
| 16 | Graphene oxide modified persimmon tannin | 50 | 8 | 180 | 404.85 |  |
| 17 | Cotton stalk-activated carbon + ZnCl_2_ | 25 | 9 | 120 | 315.45 | Deng et al.^9^ |
| 18 | Nano-zerovalent iron-Bamboo | 32 | 7.6 | 120 | 322.6 | Shaibu et al.^10^ |
| 19 | Iron/cerium modified activated carbon | 40 | 6-7 | 180 | 264.55 | Cheng et al.^11^ |
| 20 | Nanoscale manganese-bamboo | 32 | 7.6 | 120 | 263.2 | Shaibu et al.^10^ |
| 21 | Iron/cerium modified activated carbon | 30 | 6-7 | 180 | 255.76 | Cheng et al.^11^ |
| 22 | Nano-zerovalent iron | 30 | 9.5 | 1.00 | 208.33 | Arabi and Sohrabi^12^ |
| 23 | Activated carbon | 40 | 6-7 | 180 | 192.31 | Cheng et al.^11^ |
| 24 | Ball milled bamboo biochar | 25 | 5 | 1440 | 184.1 | Wang et al.^1^ |
| 26 | Activated carbon | 30 | 6-7 | 180 | 178.25 | Cheng et al.^11^ |
| 28 | Carbon nanotubes | 37 | -- | 120 | 119.71 | Shahryari et al.^13^ |
| 29 | Carbon nanotubes | 27 | -- | 120 | 109.31 |  |
| 30 | Carbon nanotubes | 17 | -- | 120 | 103.62 |  |
| 31 | SnO_2_ quantum dots decorated silica nanoparticles | 25 | 6.1 | 60.0 | 73.15 | Dutta et al.^14^ |
| 32 | Carbon nanotubes | 60 | 7 | 90.0 | 64.7 | Yao et al.^15^ |
| 33 | Carbon nanotubes | 25 | 7 | 90.0 | 46.2 |  |
| 34 | Carbon dots derived poly(4,4′-diaminodiphenylmethane | 25 | -- | 120 | 19.6 | Maruthapandi et al.^16^ |
| 35 | Sunflower oil cake-activated carbon+H_2_SO_4_ | 25 | 6 | 1440 | 16.43 | Karagöz et al.^17^ |
| 36 | Sunflower oil cake-activated carbon | 25 | 6 | 1440 | 10.21 |  |


**Fig. S1.** pH at point of zero charge (PZC) of date palm waste derived biochar (DBC), date palm waste derived biochar composite with nano zerovalent iron (nZVI-DBC) and nano carbon dots (nCD-DBC).

**Fig. S2.** Particle size distribution of **a**: date palm waste derived biochar (DBC), and **b**: date palm waste derived biochar composite with nano zerovalent iron (nZVI-DBC), and **c**: nano carbon dots (nCD-DBC).

**Fig. S3.** **a**: TGA (thermogravimetric analyses) thermograms and **b**: *R_50_* (recalcitrance potential) of date palm waste derived biochar (DBC), date palm waste derived carbon nanodots (nCD-DBC) and date palm waste derived biochar composited with nano zerovalent iron (nZVI-DBC).

**Fig. S4.** TEM (transmission electron microscope) images of date palm waste derived carbon nanodots (nCD-DBC). The diameter of the nCD-DBC particles was estimated by using Adobe Photoshop CS6 software by randomly selecting the particles in the sample^18^.

**References**

1. Wang B, Gao B, Wan Y (2019) Comparative study of calcium alginate, ball-milled biochar, and their composites on aqueous methylene blue adsorption. Environ Sci Pollut Res 26(12):11535-11541.
2. Han Q, Wang J, Goodman BA, Xie J, Liu Z (2020) High adsorption of methylene blue by activated carbon prepared from phosphoric acid treated eucalyptus residue. Powder Technol. 366:239-248.
3. Cazetta AL, Vargas AM, Nogami EM, Kunita MH, Guilherme MR, Martins AC, Silva TL, Moraes JC, Almeida VC (2011) NaOH-activated carbon of high surface area produced from coconut shell: Kinetics and equilibrium studies from the methylene blue adsorption. Chem Engi J 174:117-125.
4. Bu J, Yuan L, Zhang N, Liu D, Meng Y, Peng X (2020) High-efficiency adsorption of methylene blue dye from wastewater by a thiosemicarbazide functionalized graphene oxide composite. Diamond Relat Mater 101:107604.
5. Wan X, Zhan Y, Long Z, Zeng G, He Y (2017) Core@ double-shell structured magnetic halloysite nanotube nano-hybrid as efficient recyclable adsorbent for methylene blue removal. Chemi Engi J 330:491-504.
6. Song X, Yang P, Wu D, Zhao P, Zhao X, Yang L, Zhou Y (2020) Facile synthesis of metal-organic framework UiO-66 for adsorptive removal of methylene blue from water. Chemi Phy 531:110655.
7. Hameed BH, Din AM, Ahmad AL (2007) Adsorption of methylene blue onto bamboo-based activated carbon: kinetics and equilibrium studies. J Hazard Mater 141:819-825.
8. Wang Z, Gao M, Li X, Ning J, Zhou Z, Li G (2020) Efficient adsorption of methylene blue from aqueous solution by graphene oxide modified persimmon tannins. Mater Sci Engi C 108:110196.
9. Deng H, Yang L, Tao G, Dai J (2009) Preparation and characterization of activated carbon from cotton stalk by microwave assisted chemical activation—application in methylene blue adsorption from aqueous solution. J Hazard Mater 166:1514-1521.
10. Shaibu SE, Adekola FA, Adegoke HI, Ayanda OS (2014) A comparative study of the adsorption of methylene blue onto synthesized nanoscale zero-valent iron-bamboo and manganese-bamboo composites. Materials 7(6):4493-4507.
11. Cheng S, Zhang L, Ma A, Xia H, Peng J, Li C, Shu J (2018) Comparison of activated carbon and iron/cerium modified activated carbon to remove methylene blue from wastewater. J Environ Sci 65:92-102.
12. Arabi S, Sohrabi MR (2014) Removal of methylene blue, a basic dye, from aqueous solutions using nano-zerovalent iron. Water Sci Technol 70(1):24-31.
13. Shahryari Z, Goharrizi AS, Azadi M (2010) Experimental study of methylene blue adsorption from aqueous solutions onto carbon nano tubes. Int J Water Res Environ Engi 2(2):016-028.
14. Dutta D, Thakur D, Bahadur D (2015) SnO_2_ quantum dots decorated silica nanoparticles for fast removal of cationic dye (methylene blue) from wastewater. Chemi Engi J 281:482-490.
15. Yao Y, Xu F, Chen M, Xu Z, Zhu Z (2010) Adsorption behavior of methylene blue on carbon nanotubes. Bioresour Technol 101(9):3040-3046.
16. Maruthapandi M, Kumar VB, Gedanken A (2018) Carbon dot initiated synthesis of poly (4, 4′-diaminodiphenylmethane) and its methylene blue adsorption. ACS Omega 3(6):7061-7068.
17. Karagöz S, Tay T, Ucar S, Erdem M (2008) Activated carbons from waste biomass by sulfuric acid activation and their use on methylene blue adsorption. Bioresour Technol 99(14):6214-6222.
18. Aijaz MO, Karim MR, Alharbi HF, Alharthi NH. (2019) Novel optimised highly aligned electrospun PEI-PAN nanofibre mats with excellent wettability. Polymer 180: 121665.
